# Supplementary material for: High Rate of Mutations of Adhesion Molecules and Extracellular Matrix Glycoproteins in Patients with Adult-Onset Focal and Segmental Glomerulosclerosis
Source: Biomedicines. 2023 Jun 20;11(6):1764. doi: 10.3390/biomedicines11061764 (PMC10296603; doi:10.3390/biomedicines11061764)
Supplement: Supplementary file 1 [file biomedicines-11-01764-s001.zip › biomedicines-2420377-supplementary.pdf]

High rate of mutations of adhesion molecules and extracellular matrix glycoproteins in patients with adult onset focal and segmental glomerulosclerosis

Sara Marcos González [1], Emilio Rodrigo Calabia, Ignacio Varela, Michal Červienka, Javier Freire Salinas, and José Javier Gómez Román

[1] Pathology Department. Marqués de Valdecilla University Hospital. Institute of Research Valdecilla (IDIVAL). Santander, Spain. sara.marcos@scsalud.es

Supplemental Material Table of Contents

DNA extraction protocol (Cobas® DNA Sample Preparation Kit) …………………......……….2
Table S1. Panel of 29 genes analyzed with next generation sequencing, and distinction between nephropathic and phenocopy genes.. 2
Table S2. Total variants, silent and non-silent mutations in FSGS patients 5
Table S3. List of variants based on the UnifiedGenotyper and ACMG score 6
Tabla S4. Frequency of variants annotated found in our study.. 17

Section S1. DNA extraction protocol

1. Vortex each tissue tube with TLB/PK for 30 seconds.

2. Incubate at 56°C for 1 hour.

3. Vortex the tube for 10 seconds.

4. Incubate at 90°C for 1 hour.

5. During this incubation, prepare the necessary number of filter tubes along with their waste tubes and label them with a sample ID on the filter cap. Additionally, label a 1.5 ml Eppendorf tube for each sample to collect the final extract.

6. Remove tubes from the 90°C heat block and let them cool for 5 minutes at room temperature.

7. Perform a 3-second centrifuge pulse.

8. Add 200 µl of DNA Paraffin Binding Buffer (DNA PBB) and homogenize by aspirating/dispensing 3 times.

9. Incubate at room temperature for 10 minutes.

10. Add 100 µl of isopropanol and homogenize.

11. Transfer all the liquid from the tube by pipetting to the filter assembly plus waste tube.

12. Centrifuge at 8000g (5400rpm) for 1 minute.

13. Remove and discard the waste tube and replace it with a new one.

14. Add 500 µl of Wash Buffer I to the filter assembly plus tube.

15. Centrifuge at 8000g for 1 minute.

16. Discard the liquid from the waste tube and keep the same filter tube.

17. Add 500 µl of Wash Buffer II to the filter assembly plus tube.

18. Centrifuge at 8000g for 1 minute.

19. Remove and discard the waste tube and replace it with a new one.

20. Centrifuge at 16000g for 1 minute.

21. Remove and discard the waste tube and place the filter tube in its labeled Eppendorf tube.

22. Add 50 µl of DNA Elution Buffer (DNA EB) to the center of the filter, without touching it.

23. Incubate at room temperature for 5 minutes.

24. Centrifuge at 8000g for 1 minute to collect the extracted DNA in the Eppendorf tube.

25. Discard the filter tube.

Table S1. Panel of 29 genes analyzed with next generation sequencing, and distinction between nephropathic and phenocopy genes

Gene	*Synonym*	*Chr.*	*OMIM*	*Nephropathy/ phenocopy Genes*
ACTB actin beta	BRWS1; PS1TP5BP1	7	*102630	
ARHGDIA Rho GDP dissociation inhibitor alpha	GDIA1; NPHS8; RHOGDI; RHOGDI-1; HEL-S-47e	17	*601925	Nephrotic syndrome, type 8 [1]
CDC42 Cell division cycle 42	TKS G25K CDC42Hs	1	*116952	
CDH1 Cadherin 1	UVO; CDHE; ECAD; LCAM; Arc-1; BCDS1; CD324	16	*192090	
COL1A2 collagen type I alpha 2 chain	OI4; EDSCV; EDSARTH2	7	*120160	
ELN Elastin	WS; WBS; SVAS; ADCL1	7	*130160	
FN1 fibronectin 1	FN; CIG; FNZ; MSF; ED-B; FINC; GFND; LETS; GFND2; SMDCF	2	*135600	Glomerulopathy with fibronectin deposits [2]
FSCN1 fascin actin-bundling protein 1	HSN; SNL; p55; FAN1	7	*602689	
ICAM1 Intercellular adhesion molecule 1	BB2 CD54 P3.58	19	*147840	
ITGA5 integrin subunit alpha 5	FNRA; CD49e; VLA-5; VLA5A	12	*135620	
ITGB1 integrin subunit beta 1	CD29; FNRB; MDF2; VLAB; GPIIA; MSK12; VLA-BETA	10	*135630	
ITGB3 integrin subunit beta 3	GT; CD61; GP3A; BDPLT2; GPIIIa; BDPLT16	17	*173470	
LAMC1 laminin subunit gamma 1	LAMININ B2, FORMERLY; LAMB2, FORMERLY	1	*150290	
LPAR1 lysophosphatidic acid receptor 1	EDG2; LPA1; VZG1; GPR26; edg-2; vzg-1; Gpcr26; Mrec1.3; rec.1.3	9	*602282	
LPAR2 lysophosphatidic acid receptor 2	EDG4; LPA2; EDG-4; LPA-2	19	*605110	
LPAR3 lysophosphatidic acid receptor 3	EDG7; GPCR; LPA3; Edg-7; LP-A3; HOFNH30; RP4-678I3	1	*605106	
LPAR4 lysophosphatidic acid receptor 4	LPA4; P2Y9; GPR23; P2RY9; P2Y5-LIKE	X	*300086	
LPAR5 lysophosphatidic acid receptor 5	GPR92, GPR93, KPG_010, LPA5	12	*606926	
LPAR6	ARWH1, HYPT8, LAH3,	13	*609239	

lysophosphatidic acid receptor 6	LPA-6, P2RY5, P2Y5			
PTK2<br>protein tyrosine kinase 2	FAK; FADK; FAK1; FRNK; PPP1R71; p125FAK; pp125FAK	8	*600758	
RHOA<br>Ras homolog family member A	ARHA; ARH12; RHO12; EDFAOB; RHOH12	3	*165390	
RHOC<br>Ras homolog family member C	H9<br>ARH9<br>ARHC<br>RHOH9	1	*165380	
ROCK2<br>Rho associated coiled-coil containing protein kinase 2	ROCK-II	2	*604002	
SELE<br>selectin E	ELAM; ESEL; CD62E; ELAM1; LECAM2	1	*131210	IgA nephropathy [3]
TNC<br>Tenascin C	GP; JI; TN; HXB; GMEM; TN-C; DFNA56; 150-225	9	*187380	Diverse glomerulopathies [4]
TPM1<br>Tropomyosin 1	CMH3;<br>TMSA;<br>CMD1Y; LVNC9;<br>C15orf13;<br>HEL-S-265; HTM-alpha	15	*191010	
TRAM1<br>Translocation associated membrane protein 1	TRAM<br>PNAS8<br>TRAMP	8	*605190	
VCAM1<br>vascular cell adhesion molecule 1	CD106; INCAM-100	1	*192225	
WASF3<br>WASP family member 3	SCAR3<br>WAVE3<br>Brush-1	13	*605068	

Table S2. Total variants, silent and non-silent mutations in FSGS patients

Gene	Total variants	FSGS Non-Silent variants
ACTB	17	0
ARHGDIA	2	0
CDC42	6	0
CDH1	23	10
COL1A2	23	21
ELN	20	18
FN1	23	23
FSCN1	9	0
ICAM1	19	17
ITGA5	23	0
ITGB1	23	3
ITGB3	18	18
LAMC1	21	20
LPAR1	11	0
LPAR2	23	0
LPAR3	5	5
LPAR4	9	9
LPAR5	0	0
LPAR6	11	10
PTK2	23	0
RHOA	0	0
RHOC	21	0
ROCK2	22	14
SELE	9	9
TNC	23	23
TPM1	23	0
TRAM1	23	0
VCAM1	23	2
WASF3	17	1

Table S3. List of variants based on the UnifiedGenotyper and ACMG score

Patient	*Mutations*	*Non-silent mutations*	*Gene*	*ID*	*Nucleotide change*	*Amino acid change*	*Zygosity*	*Type of Mutation*	*Classify Variant **VarSome***
Case 1	87	19	*TNC*	rs113301777	c.3739 C>A	p.Leu1247Ile	*Het*	Missense	Benign
				rs1061494	c.2039 A>G	p.Gln680Arg			
				rs13321	c.6022 G>C	p.Glu2008Gln	*Hom*		
				rs1757095	c.1616 A>G	p.Gln539Arg			
			ICAM1	rs5498	c.1405 A>G	p.Lys469Glu	*Het*	Missense	Benign
			ROCK2	rs2230774	c.1292 C>A	p.Thr431Asn	*Het*	Missense	Benign
			ITGB1	-	c.1807 A>T	p.Asn603Tyr	*Het*	Missense	VUS
			ITGB3	rs15908	c.1108 A>C	p.Val370Leu	*Het*	Missense	Benign
			LPAR4	-	c.260 T>C	p.Leu87Pro	*Het*	Missense	VUS
			ELN	rs2071307	c.1264 G>A	p.Gly422Ser	*Het*	Missense	Benign
			COL1A2	rs42524	c.1645 C>G	p.Pro549Ala	*Hom*	Missense	Benign
			LAMC1	-	c.148 T>C	p.Cys50Arg	*Het*	Missense	VUS
				rs20563	c.1372 A>G	p.Ile458Val			Benign
				rs20558	c.2663 T>C	p.Leu888Pro			
			CDH1	-	c.499 G>T	p.Glu167*	*Het*	Nonsense	Pathogenic
				-	c.500 A>G	p.Glu167Gly		Missense	Likely Benign
			FN1	rs1250209	c.6415 G>A	p.Val2139Ile	*Hom*	Missense	Benign
				rs2577301	c.2449 A>C	p.Thr817Pro			
				rs1250259	c.44 A>T	p.Gln15Leu			
Case 2	103	20	*TNC*	rs13321	c.6022 G>C	p.Glu2008Gln	*Hom*	Missense	Benign
				rs1061494	c.2039 A>G	p.Gln680Arg	*Het*		
				rs1757095	c.1616 A>G	p.Gln539Arg	*Hom*		
			SELE	rs5368	c.1402 C>T	p.His468Tyr	*Het*	Missense	Benign
			ICAM1	rs1799969	c.721 G>A	p.Gly241Arg	*Het*	Missense	Benign
				rs5498	c.1405 A>G	p.Lys469Glu			
			ROCK2	rs2230774	c.1292 C>A	p.Thr431Asn	*Het*	Missense	Benign
			ITGB3	rs15908	c.1108 A>C	p.Val370Leu	*Het*	Missense	-
				rs121918449	c.1199 G>A	p.Cys400Tyr			Likely Pathogenic
			LPAR4	-	c.260 T>C	p.Leu87Pro	*Het*	Missense	VUS
			ELN	rs17855988	c.1741 G>C	p.Gly581Arg	*Het*	Missense	Benign
			COL1A2	-	c.1015 A>C	p.Thr339Pro	*Het*	Missense	VUS
				rs42524	c.1645 C>G	p.Pro549Ala	*Hom*		Benign

			LAMC1	rs20563	c.1372 A>G	p.Ile458Val	*Hom*	Missense	Benign
				rs20558	c.2663 T>C	p.Leu888Pro			
			CDH1	-	c.499 G>T	p.Glu167*	*Het*	Nonsense	Pathogenic
				-	c.500 A>G	p.Glu167Gly		Missense	Likely Benign
			FN1	rs1250209	c.6415 G>A	p.Val2139Ile	*Hom*	Missense	Benign
				rs2577301	c.2449 A>C	p.Thr817Pro			
				rs1250259	c.44 A>T	p.Gln15Leu			
Case 3	91	18	*TNC*	rs13321	c.6022 G>C	p.Glu2008Gln	*Het*	Missense	Benign
				rs2104772	c.5029 A>T	p.Ile1677Leu			
				rs1061494	c.2039 A>G	p.Gln680Arg			
				rs1757095	c.1616 A>G	p.Gln539Arg	*Hom*		
			SELE	rs5361	c.445 A>C	p.Ser149Arg	*Het*	Missense	Benign
			ROCK2	rs2230774	c.1292 C>A	p.Thr431Asn	*Het*	Missense	Benign
			ITGB3	rs15908	c.1108 A>C	p.Val370Leu	*Het*	Missense	-
			LPAR3	-	c.524 C>G	p.Ala175Gly	*Het*	Missense	VUS
			LPAR4	-	c.260 T>C	p.Leu87Pro	*Het*	Missense	VUS
			ELN	rs2071307	c.1264 G>A	p.Gly422Ser	*Hom*	Missense	Benign
				rs17855988	c.1741 G>C	p.Gly581Arg			
				rs41511151	c.2078 G>A	p.Gly693Asp	*Het*		
			COL1A2	-	c.1015 A>C	p.Thr339Pro	*Het*	Missense	VUS
				rs42524	c.1645 C>G	p.Pro549Ala			Benign
			CDH1	-	c.500 A>G	p.Glu167Gly	*Het*	Missense	Likely Benign
			FN1	rs1250209	c.6415 G>A	p.Val2139Ile	*Het*	Missense	Benign
				rs2577301	c.2449 A>C	p.Thr817Pro	*Hom*		
				rs1250259	c.44 A>T	p.Gln15Leu	*Het*		
Case 4	86	16	*TNC*	rs13321	c.6022 G>C	p.Glu2008Gln	*Hom*	Missense	Benign
				rs1757095	c.1616 A>G	p.Gln539Arg			
			ICAM1	rs5498	c.1405 A>G	p.Lys469Glu	*Hom*	Missense	Benign
			ITGB3	rs15908	c.1108 A>C	p.Val370Leu	*Het*	Missense	-
				rs367659742	c.2351 C>T	p.Thr784Met			VUS
			LPAR4	rs148919808	c.133 G>A	p.Val45Ile	*Hom*	Missense	Likely Benign
				-	c.260 T>C	p.Leu87Pro	*Het*		VUS
			ELN	-	c.1435 G>A	p.Val479Met	*Het*	Missense	Likely Benign
			COL1A2	-	c.1015 A>C	p.Thr339Pro	*Het*	Missense	VUS
				rs42524	c.1645 C>G	p.Pro549Ala			Benign
			LAMC1	rs20563	c.1372 A>G	p.Ile458Val	*Het*	Missense	Benign

				rs20558	c.2663 T>C	p.Leu888Pro			
			CDH1	-	c.500 A>G	p.Glu167Gly	*Het*	Missense	Likely Benign
			FN1	rs1250209	c.6415 G>A	p.Val2139Ile	*Hom*	Missense	Benign
				rs2577301	c.2449 A>C	p.Thr817Pro			
				rs1250259	c.44 A>T	p.Gln15Leu			
Case 5	105	20	*TNC*	rs13321	c.6022 G>C	p.Glu2008Gln	*Hom*	Missense	Benign
				rs2104772	c.5029 A>T	p.Ile1677Leu	*Het*		
				rs1061494	c.2039 A>G	p.Gln680Arg			
				rs1757095	c.1616 A>G	p.Gln539Arg	*Hom*		
			ICAM1	rs5498	c.1405 A>G	p.Lys469Glu	*Het*	Missense	Benign
			ITGB3	rs15908	c.1108 A>C	p.Val370Leu	*Het*	Missense	-
			LPAR4	-	c.259 C>G	p.Leu87Val	*Het*	Missense	VUS
				-	c.260 T>C	p.Leu87Pro			
			LPAR6	-	c.998 T>C	p.Leu333Ser	*Het*	Missense	VUS
			ELN	-	c.1435 G>A	p.Val479Met	*Het*	Missense	Likely Benign
				rs17855988	c.1741 G>C	p.Gly581Arg			Benign
			COL1A2	-	c.1015 A>C	p.Thr339Pro	*Het*	Missense	VUS
				rs42524	c.1645 C>G	p.Pro549Ala	*Hom*		Benign
			LAMC1	rs20563	c.1372 A>G	p.Ile458Val	*Hom*	Missense	Benign
				rs20558	c.2663 T>C	p.Leu888Pro			
			CDH1	-	c.499 G>T	p.Glu167*	*Het*	Nonsense	Pathologic
				-	c.500 A>G	p.Glu167Gly		Missense	Likely Benign
			FN1	rs1250209	c.6415 G>A	p.Val2139Ile	*Het*	Missense	Benign
				rs2577301	c.2449 A>C	p.Thr817Pro	*Hom*		
				rs1250259	c.44 A>T	p.Gln15Leu	*Het*		
Case 6	100	17	*TNC*	rs13321	c.6022 G>C	p.Glu2008Gln	*Hom*	Missense	Benign
				rs2104772	c.5029 A>T	p.Ile1677Leu	*Het*		
				rs1757095	c.1616 A>G	p.Gln539Arg	*Hom*		
			SELE	rs5368	c.1402 C>T	p.His468Tyr	*Het*	Missense	Benign
			ICAM1	rs5498	c.1405 A>G	p.Lys469Glu	*Het*	Missense	Benign
			ROCK2	rs2230774	c.1292 C>A	p.Thr431Asn	*Hom*	Missense	Benign
			ITGB3	rs15908	c.1108 A>C	p.Val370Leu	*Het*	Missense	-
			LPAR3	-	c.524 C>G	p.Ala175Gly	*Het*	Missense	VUS
			LPAR4	-	c.260 T>C	p.Leu87Pro	*Het*	Missense	VUS
			ELN	rs2071307	c.1264 G>A	p.Gly422Ser	*Het*	Missense	Benign
				-	c.1435 G>A	p.Val479Met			Likely Benign

			COL1A2	rs42524	c.1645 C>G	p.Pro549Ala	*Hom*	Missense	Benign
			LAMC1	rs20563	c.1372 A>G	p.Ile458Val	*Hom*	Missense	Benign
				rs20558	c.2663 T>C	p.Leu888Pro			
			FN1	rs1250209	c.6415 G>A	p.Val2139Ile	*Het*	Missense	Benign
				rs2577301	c.2449 A>C	p.Thr817Pro	*Hom*		
				rs1250259	c.44 A>T	p.Gln15Leu	*Het*		
Case 7	97	18	*TNC*	rs13321	c.6022 G>C	p.Glu2008Gln	*Hom*	Missense	Benign
				rs113301777	c.3739 C>A	p.Leu1247Ile	*Het*		
				rs1061494	c.2039 A>G	p.Gln680Arg	*Hom*		
				rs1757095	c.1616 A>G	p.Gln539Arg			
			SELE	rs5361	c.445 A>C	p.Ser149Arg	*Het*	Missense	Benign
			ROCK2	rs2230774	c.1292 C>A	p.Thr431Asn	*Hom*	Missense	Benign
			ITGB3	rs15908	c.1108 A>C	p.Val370Leu	*Hom*	Missense	-
				rs121918449	c.1199 G>A	p.Cys400Tyr	*Het*		Likely Pathogenic
			LPAR3	-	c.524 C>G	p.Ala175Gly	*Het*	Missense	VUS
			ELN	rs2071307	c.1264 G>A	p.Gly422Ser	*Hom*	Missense	Benign
				-	c.1435 G>A	p.Val479Met	*Het*		Likely Benign
			COL1A2	rs42524	c.1645 C>G	p.Pro549Ala	*Hom*	Missense	Benign
			LAMC1	rs34995260	c.3796 G>A	p.Glu1266Lys	*Het*	Missense	Likely Benign
			CDH1	-	c.499 G>T	p.Glu167*	*Het*	Nonsense	Pathogenic
				-	c.500 A>G	p.Glu167Gly		Missense	Likely Benign
			FN1	rs1250209	c.6415 G>A	p.Val2139Ile	*Het*	Missense	Benign
				rs2577301	c.2449 A>C	p.Thr817Pro	*Hom*		
				rs1250259	c.44 A>T	p.Gln15Leu			
Case 8	84	13	*TNC*	rs13321	c.6022 G>C	p.Glu2008Gln	*Hom*	Missense	Benign
				rs2104772	c.5029 A>T	p.Ile1677Leu	*Het*		
				rs1138545	c.3197 G>A	p.Arg1066His			
				rs1757095	c.1616 A>G	p.Gln539Arg	*Hom*		
			ICAM1	rs5498	c.1405 A>G	p.Lys469Glu	*Het*	Missense	Benign
			ITGB3	rs15908	c.1108 A>C	p.Val370Leu	*Hom*	Missense	-
			COL1A2	rs42524	c.1645 C>G	p.Pro549Ala	*Het*	Missense	Benign
			LAMC1	rs20563	c.1372 A>G	p.Ile458Val	*Het*	Missense	Benign
				rs20558	c.2663 T>C	p. Leu888Pro			
			CDH1	-	c.500 A>G	p.Glu167Gly	*Het*	Missense	Likely Benign
			FN1	rs1250209	c.6415 G>A	p.Val2139Ile	*Het*	Missense	Benign
				rs2577301	c.2449 A>C	p.Thr817Pro	*Hom*		

				rs1250259	c.44 A>T	p.Gln15Leu			
Case 9	97	17	*TNC*	rs13321	c.6022 G>C	p.Glu2008Gln	*Hom*	Missense	Benign
				rs1061494	c.2039 A>G	p.Gln680Arg	*Het*		
				rs1757095	c.1616 A>G	p.Gln539Arg	*Hom*		
			ROCK2	rs2230774	c.1292 C>A	p.Thr431Asn	*Het*	Missense	Benign
			ITGB3	rs5918	c.176 T>C	p.Leu59Pro	*Het*	Missense	Benign
				rs15908	c.1108 A>C	p.Val370Leu			-
			LPAR4	-	c.260 T>C	p.Leu87Pro	*Het*	Missense	VUS
			LPAR6	-	c.227 A>T	p.Tyr76Phe	*Het*	Missense	VUS
			COL1A2	-	c.1015 A>C	p.Thr339Pro	*Het*	Missense	VUS
				rs42524	c.1645 C>G	p.Pro549Ala			Benign
			LAMC1	rs20563	c.1372 A>G	p.Ile458Val	*Het*	Missense	Benign
				rs20558	c.2663 T>C	p. Leu888Pro			
			CDH1	-	c.499 G>T	p.Glu167*	*Het*	Nonsense	Pathogenic
				-	c.500 A>G	p.Glu167Gly		Missense	Likely Benign
			FN1	rs1250209	c.6415 G>A	p.Val2139Ile	*Het*	Missense	Benign
				rs2577301	c.2449 A>C	p.Thr817Pro	*Hom*		
				rs1250259	c.44 A>T	p.Gln15Leu			
Case 10	98	22	*TNC*	rs13321	c.6022 G>C	p.Glu2008Gln	*Hom*	Missense	Benign
				rs2274750	c.5341 G>A	p.Ala1781Thr	*Het*		
				rs2104772	c.5029 A>T	p.Ile1677Leu			
				rs1061494	c.2039 A>G	p.Gln680Arg	*Hom*		
				rs1757095	c.1616 A>G	p.Gln539Arg			
			ICAM1	rs5498	c.1405 A>G	p.Lys469Glu	*Het*	Missense	Benign
			ITGB1	-	c.1807 A>T	p.Asn603Tyr	*Het*	Missense	VUS
			LPAR3	-	c.524 C>G	p.Ala175Gly	*Het*	Missense	VUS
			LPAR4	-	c.259 C>G	p.Leu87Val	*Het*	Missense	VUS
				-	c.260 T>C	p.Leu87Pro			
			LPAR6	-	c.227 A>T	p.Tyr76Phe	*Het*	Missense	VUS
			ELN	rs2071307	c.1264 G>A	p.Gly422Ser	*Het*	Missense	Benign
				-	c.1435 G>A	p.Val479Met			Likely Benign
			COL1A2	-	c.1015 A>C	p.T339Pro	*Het*	Missense	VUS
				rs42524	c.1645 C>G	p.Pro549Ala	*Hom*		Benign
			LAMC1	rs20563	c.1372 A>G	p.Ile458Val	*Hom*	Missense	Benign
				rs20558	c.2663 T>C	p. Leu888Pro			
				rs61749262	c.3614 A>G	p.Asn1205Ser	*Het*		

			CDH1	-	c.500 A>G	p.Glu167Gly	*Het*	Missense	Likely Benign
			FN1	rs1250209	c.6415 G>A	p.Val2139Ile	*Hom*	Missense	Benign
				rs2577301	c.2449 A>C	p.Thr817Pro			
				rs1250259	c.44 A>T	p.Gln15Leu			
Case 11	95	18	*TNC*	rs1061494	c.2039 A>G	p.Gln680Arg	*Het*	Missense	Benign
				rs1757095	c.1616 A>G	p.Gln539Arg	*Hom*		
			SELE	rs5361	c.445 A>C	p.Ser149Arg	*Het*	Missense	Benign
			ICAM1	rs5498	c.1405 A>G	p.Lys469Glu	*Hom*	Missense	Benign
				rs376526495	c.1589 C>T	p.Thr530Met	*Het*		Likely Benign
			ROCK2	rs2230774	c.1292 C>A	p.Thr431Asn	*Hom*	Missense	Benign
			ITGB3	rs15908	c.1108 A>C	p.Val370Leu	*Hom*	Missense	-
			LPAR3	-	c.524 C>G	p.Ala175Gly	*Het*	Missense	VUS
			LPAR4	-	c.259 C>G	p.Leu87Val	*Het*	Missense	VUS
				-	c.260 T>C	p.Leu87Pro			
			ELN	rs2071307	c.1264 G>A	p.Gly422Ser	*Hom*	Missense	Benign
				-	c.1435 G>A	p.Val479Met	*Het*		Likely Benign
				rs17855988	c.1741 G>C	p.Gly581Arg			Benign
			LAMC1	rs20563	c.1372 A>G	p.Ile458Val	*Hom*	Missense	Benign
				rs20558	c.2663 T>C	p. Leu888Pro			
			FN1	rs1250209	c.6415 G>A	p.Val2139Ile	*Het*	Missense	Benign
				rs2577301	c.2449 A>C	p.Thr817Pro	*Hom*		
				rs1250259	c.44 A>T	p.Gln15Leu			
Case 12	97	15	*TNC*	rs13321	c.6022 G>C	p.Glu2008Gln	*Het*	Missense	Benign
				rs2104772	c.5029 A>T	p.Ile1677Leu			
				rs1138545	c.3197 G>A	p.Arg1066His			
				rs1061494	c.2039 A>G	p.Gln680Arg			
				rs1757095	c.1616 A>G	p.Gln539Arg	*Hom*		
			ROCK2	rs2230774	c.1292 C>A	p.Thr431Asn	*Het*	Missense	Benign
			ITGB3	rs15908	c.1108 A>C	p.V370L	*Het*	Missense	-
			LPAR6	-	c.227 A>T	p.Y76F	*Het*	Missense	VUS
			ELN	rs2071307	c.1264 G>A	p.Gly422Ser	*Het*	Missense	Benign
			COL1A2	rs42524	c.1645 C>G	p.Pro549Ala	*Hom*	Missense	Benign
			LAMC1	rs20563	c.1372 A>G	p.Ile458Val	*Het*	Missense	Benign
				rs20558	c.2663 T>C	p. Leu888Pro			
			FN1	rs1250209	c.6415 G>A	p.V2139I	*Hom*	Missense	Benign
				rs2577301	c.2449 A>C	p.Thr817Pro			

				rs1250259	c.44 A>T	p.Gln15Leu			
Case 13	93	18	*TNC*	-	Exon 28 - 2 A>T	-	*Het*	Essential splice	-
				rs13321	c.6022 G>C	p.Glu2008Gln	*Hom*	Missense	Benign
				rs2104772	c.5029 A>T	p.Ile1677Leu	*Het*		
				rs1061494	c.2039 A>G	p.Gln680Arg			
				rs1757095	c.1616 A>G	p.Gln539Arg	*Hom*		
			ICAM1	rs1801714	c.1055 C>T	p.Pro352Leu	*Het*	Missense	Benign
				rs5498	c.1405 A>G	p.Lys469Glu			
			ROCK2	rs2230774	c.1292 C>A	p.Thr431Asn	*Het*	Missense	Benign
			ELN	rs2071307	c.1264 G>A	p.Gly422Ser	*Het*	Missense	Benign
				rs17855988	c.1741 G>C	p.Gly581Arg			
			COL1A2	-	c.1015 A>C	p.Thr339Pro	*Het*	Missense	VUS
				rs42524	c.1645 C>G	p.Pro549Ala	*Hom*		Benign
			LAMC1	rs20563	c.1372 A>G	p.Ile458Val	*Het*	Missense	Benign
				rs20558	c.2663 T>C	p.Leu888Pro			
				rs61749262	c.3614 A>G	p.Asn1205Ser	*Het*		
			FN1	rs1250209	c.6415 G>A	p.Val2139Ile	*Hom*	Missense	Benign
				rs2577301	c.2449 A>C	p.Thr817Pro			
				rs1250259	c.44 A>T	p.Gln15Leu			
Case 14	86	20	*TNC*	rs13321	c.6022 G>C	p.Glu2008Gln	*Hom*	Missense	Benign
				rs2104772	c.5029 A>T	p.Ile1677Leu	*Het*		
				rs113301777	c.3739 C>A	p.Leu1247Ile			
				rs1138545	c.3197 G>A	p.Arg1066His			
				rs1061494	c.2039 A>G	p.Gln680Arg			
				rs1757095	c.1616 A>G	p.Gln539Arg	*Hom*		
			SELE	rs5368	c.1402 C>T	p.His468Tyr	*Het*	Missense	Benign
			ICAM1	rs1801714	c.1055 C>T	p.Pro352Leu	*Het*	Missense	Benign
				rs5498	c.1405 A>G	p.Lys469Glu	*Hom*		
			ROCK2	rs2230774	c.1292 C>A	p.Thr431Asn	*Het*	Missense	Benign
			ITGB3	rs5918	c.176 T>C	p.Leu59Pro	*Het*	Missense	Benign
				rs15908	c.1108 A>C	p.Val370Leu			-
			LPAR6	-	c.227 A>T	p.Tyr76Phe	*Het*	Missense	VUS
			ELN	rs2071307	c.1264 G>A	p.Gly422Ser	*Hom*	Missense	Benign
			COL1A2	rs42524	c.1645 C>G	p.Pro549Ala	*Het*	Missense	Benign
			LAMC1	rs20563	c.1372 A>G	p.Ile458Val	*Het*	Missense	Benign
				rs20558	c.2663 T>C	p. Leu888Pro			

			FN1	rs1250209	c.6415 G>A	p.Val2139Ile	*Het*	Missense	Benign
				rs2577301	c.2449 A>C	p.Thr817Pro	*Hom*		
				rs1250259	c.44 A>T	p.Gln15Leu			
Case 15	84	9	*TNC*	rs13321	c.6022 G>C	p.Glu2008Gln	*Hom*	Missense	Benign
				rs1061494	c.2039 A>G	p.Gln680Arg			
				rs1757095	c.1616 A>G	p.Gln539Arg			
			SELE	rs5361	c.445 A>C	p.Ser149Arg	*Het*	Missense	Benign
			ITGB3	rs15908	c.1108 A>C	p.Val370Leu	*Het*	Missense	-
			ELN	rs2071307	c.1264 G>A	p.Gly422Ser	*Het*	Missense	Benign
			COL1A2	rs42524	c.1645 C>G	p.Pro549Ala	*Hom*	Missense	Benign
			FN1	rs1250209	c.6415 G>A	p.Val2139Ile	*Het*	Missense	Benign
				rs2577301	c.2449 A>C	p.Thr817Pro	*Hom*		
Case 16	90	13	*TNC*	rs13321	c.6022 G>C	p.Glu2008Gln	*Hom*	Missense	Benign
				rs1061494	c.2039 A>G	p.Gln680Arg			
				rs1757095	c.1616 A>G	p.Gln539Arg			
			ICAM1	rs5498	c.1405 A>G	p.Lys469Glu	*Het*	Missense	Benign
			ROCK2	rs2230774	c.1292 C>A	p.Thr431Asn	*Het*	Missense	Benign
			LPAR6	-	c.998 T>C	p.Leu333Ser	*Het*	Missense	VUS
			ELN	-	c.1435 G>A	p.Val479Met	*Het*	Missense	Likely Benign
				rs17855988	c.1741 G>C	p.Gly581Arg			Benign
			LAMC1	rs20563	c.1372 A>G	p.Ile458Val	*Het*	Missense	Benign
				rs20558	c.2663 T>C	p. Leu888Pro			
			FN1	rs1250209	c.6415 G>A	p.Val2139Ile	*Het*	Missense	Benign
				rs2577301	c.2449 A>C	p.Thr817Pro	*Hom*		
				rs1250259	c.44 A>T	p.Gln15Leu			
Case 17	106	17	*TNC*	rs13321	c.6022 G>C	p.Glu2008Gln	*Hom*	Missense	Benign
				rs2104772	c.5029 A>T	p.Ile1677Leu	*Het*		
				-	c.4241 G>C	p.Arg1414Thr			Likely Benign
				rs1138545	c.3197 G>A	p.Arg1066His			Benign
				rs1757095	c.1616 A>G	p.Gln539Arg	*Hom*		
			SELE	rs5368	c.1402 C>T	p.His468Tyr	*Hom*	Missense	Benign
			ICAM1	rs5498	c.1405 A>G	p.Lys469Glu	*Het*	Missense	Benign
			ROCK2	rs2230774	c.1292 C>A	p.Thr431Asn	*Het*	Missense	Benign
			ITGB3	rs5918	c.176 T>C	p.Leu59Pro	*Het*	Missense	Likely Benign
				rs15908	c.1108 A>C	p.Val370Leu			-
			COL1A2	-	c.1015 A>C	p.Thr339Pro	*Het*	Missense	VUS

				rs42524	c.1645 C>G	p.Pro549Ala			Benign
			LAMC1	rs20563	c.1372 A>G	p.Ile458Val	*Hom*	Missense	Benign
				rs148690613	c.2053 A>G	p.Thr685Ala	*Het*		Likely Benign
				rs20558	c.2663 T>C	p. Leu888Pro	*Hom*		Benign
			FN1	rs1250209	c.6415 G>A	p.Val2139Ile	*Het*	Missense	Benign
				rs2577301	c.2449 A>C	p.Thr817Pro	*Hom*		
Case 18	99	14	*TNC*	rs13321	c.6022 G>C	p.Glu2008Gln	*Hom*	Missense	Benign
				rs2104772	c.5029 A>T	p.Ile1677Leu			
				rs1757095	c.1616 A>G	p.Gln539Arg			
			ITGB3	rs5918	c.176 T>C	p.Leu59Pro	*Het*	Missense	Benign
				rs15908	c.1108 A>C	p.Val370Leu	*Hom*		-
			LPAR6	-	c.227 A>T	p.Tyr76Phe	*Het*	Missense	VUS
			ELN	rs2071307	c.1264 G>A	p.Gly422Ser	*Hom*	Missense	Benign
				rs17855988	c.1741 G>C	p.Gly581Arg	*Het*		
			COL1A2	rs42524	c.1645 C>G	p.Pro549Ala	*Het*	Missense	Benign
			LAMC1	rs20563	c.1372 A>G	p.Ile458Val	*Het*	Missense	Benign
				rs20558	c.2663 T>C	p. Leu888Pro			
			FN1	rs1250209	c.6415 G>A	p.Val2139Ile	*Het*	Missense	Benign
				rs2577301	c.2449 A>C	p.Thr817Pro	*Hom*		
				rs1250259	c.44 A>T	p.Gln15Leu			
Case 19	101	17	*TNC*	rs13321	c.6022 G>C	p.Glu2008Gln	*Het*	Missense	Benign
				rs61734387	c.5093 G>C	p.Arg1698Pro			Benign
				rs113301777	c.3739 C>A	p.Leu1247Ile			Benign
				rs1061494	c.2039 A>G	p.Gln680Arg			
				rs1757095	c.1616 A>G	p.Gln539Arg	*Hom*		
			WASF3	-	c.934 G>C	p.Ala312Pro	*Het*	Missense	VUS
			ICAM1	rs5498	c.1405 A>G	p.Lys469Glu	*Het*	Missense	Benign
			ROCK2	rs2230774	c.1292 C>A	p.Thr431Asn	*Het*	Missense	Benign
			ITGB3	rs5918	c.176 T>C	p.Leu59Pro	*Het*	Missense	Benign
				rs15908	c.1108 A>C	p.Val370Leu			-
			ELN	rs17855988	c.1741 G>C	p.Gly581Arg	*Het*	Missense	Benign
			COL1A2	rs42524	c.1645 C>G	p.Pro549Ala	*Hom*	Missense	Benign
			LAMC1	rs20563	c.1372 A>G	p.Ile458Val	*Hom*	Missense	Benign
				rs20558	c.2663 T>C	p. Leu888Pro			
			FN1	rs1250209	c.6415 G>A	p.Val2139Ile	*Hom*	Missense	Benign
				rs2577301	c.2449 A>C	p.Thr817Pro			

				rs1250259	c.44 A>T	p.Gln15Leu	*Het*		
Case 20	79	15	*TNC*	rs13321	c.6022 G>C	p.Glu2008Gln	*Het*	Missense	Benign
				rs61734387	c.5093 G>C	p.Arg1698Pro			
				rs2104772	c.5029 A>T	p.Ile1677Leu			
				rs1061494	c.2039 A>G	p.Gln680Arg	*Hom*		
				rs1757095	c.1616 A>G	p.Gln539Arg	*Het*		
			ICAM1	rs5498	c.1405 A>G	p.Lys469Glu	*Het*	Missense	Benign
			ROCK2	rs2230774	c.1292 C>A	p.Thr431Asn	*Hom*	Missense	Benign
			ELN	rs2071307	c.1264 G>A	p.Gly422Ser	*Hom*	Missense	Benign
				rs41511151	c.2078 G>A	p.Gly693Asp	*Het*		
			COL1A2	-	c.1015 A>C	p.Thr339Pro	*Het*	Missense	VUS
			LAMC1	rs20563	c.1372 A>G	p.Ile458Val	*Hom*	Missense	Benign
				rs20558	c.2663 T>C	p. Leu888Pro			
			FN1	rs1250209	c.6415 G>A	p.Val2139Ile	*Hom*	Missense	Benign
				rs2577301	c.2449 A>C	p.Thr817Pro			
				rs1250259	c.44 A>T	p.Gln15Leu	*Het*		
Case 21	87	14	*TNC*	rs13321	c.6022 G>C	p.Glu2008Gln	*Het*	Missense	Benign
				rs2104772	c.5029 A>T	p.Ile1677Leu			
				rs1757095	c.1616 A>G	p.Gln539Arg			
			SELE	rs5366	c.1261 G>C	p.Glu421Gln	*Het*	Missense	Benign
			VCAM1	rs3783613	c.1052 G>C	p.Gly351Ala	*Het*	Missense	Benign
			ICAM1	-	Exon 3 + 1 G>A	-	*Het*	Essential splice	-
			ITGB3	rs15908	c.1108 A>C	p.Val370Leu	*Hom*	Missense	-
				rs73322311	c.2173 C>T	p.Ala725Ser	*Het*	Missense	-
			LPAR6	-	c.227 A>T	p.Tyr76Phe	*Het*	Missense	VUS
			COL1A2	rs42524	c.1645 C>G	p.Pro549Ala	*Hom*	Missense	Benign
			FN1	rs1250209	c.6415 G>A	p.Val2139Ile	*Hom*	Missense	Benign
				rs2577301	c.2449 A>C	p.Thr817Pro			
				rs1250259	c.44 A>T	p.Gln15Leu	*Het*		
				rs115937626	c.40 G>C	p.Val14Leu			
Case 22	95	16	*TNC*	-	Exon 28 - 2 A>T		*Het*	Essential splice	-
				rs13321	c.6022 G>C	p.Glu2008Gln	*Hom*	Missense	Benign
				rs1061494	c.2039 A>G	p.Gln680Arg	*Het*		
				rs1757095	c.1616 A>G	p.Gln539Arg	*Hom*		
			VCAM1	rs3783615	c.1960 A>T	p.Ile654Leu	*Het*	Missense	Benign
			ICAM1	rs5491	c.167 A>T	p.Lys56Met	*Het*	Missense	Benign

			ITGB3	rs15908	c.1108 A>C	p.Val370Leu	*Het*	Missense	-
			LPAR6	-	c.227 A>T	p.Tyr76Phe	*Het*	Missense	VUS
			COL1A2	-	c.1015 A>C	p.Thr339Pro	*Het*	Missense	VUS
				rs42524	c.1645 C>G	p.Pro549Ala	*Hom*		Benign
			LAMC1	rs20563	c.1372 A>G	p.Ile458Val	*Het*	Missense	Benign
				rs20558	c.2663 T>C	p. Leu888Pro			
			CDH1	rs33935154	c.1849 G>A	p.Ala617Thr	*Het*	Missense	Benign
			FN1	rs1250209	c.6415 G>A	p.Val2139Ile	*Hom*	Missense	Benign
				rs2577301	c.2449 A>C	p.Thr817Pro			
				rs1250259	c.44 A>T	p.Gln15Leu	*Het*		
Case 23	86	14	*TNC*	rs13321	c.6022 G>C	p.Glu2008Gln	*Het*	Missense	Benign
				rs2104772	c.5029 A>T	p.Ile1677Leu			
				rs1061494	c.2039 A>G	p.Gln680Arg			
				rs1757095	c.1616 A>G	p.Gln539Arg	*Hom*		
			ICAM1	rs5498	c.1405 A>G	p.Lys469Glu	*Het*	Missense	Benign
			ITGB1	-	c.1807 A>T	p.Asn603Tyr	*Het*	Missense	VUS
			LPAR6	-	c.227 A>T	p.Tyr76Phe	*Het*	Missense	VUS
			ELN	-	c.1435 G>A	p.Val479Met	*Het*	Missense	Likely Benign
			COL1A2	rs42524	c.1645 C>G	p.Pro549Ala	*Hom*	Missense	Benign
			LAMC1	rs20563	c.1372 A>G	p.Ile458Val	*Hom*	Missense	Benign
				rs20558	c.2663 T>C	p. Leu888Pro			
			FN1	rs1250209	c.6415 G>A	p.Val2139Ile	*Hom*	Missense	Benign
				rs2577301	c.2449 A>C	p.Thr817Pro			
				rs1250259	c.44 A>T	p.Gln15Leu			

Hom, homozygous; Het, heterozygous; VUS, Variant of Uncertain Significance

Tabla S4. Frequency of variants annotated found in our study

Gene	ID	Nucleotide change	Frequency in study patients (n=23)	Frequency in study control group (n=16)	Frequency in European population (dbSNP, 1000Genomes)
CDH1	rs33935154	c.1849 G>A	(1) 0.0435	0	0.0010
COL1A2	rs42524	c.1645 C>G	(20) 0.8696	(12) 0.75	0.7604
ELN	rs2071307	c.1264 G>A	(12) 0.5217	(7) 0.4375	0.4205
	rs17855988	c.1741 G>C	(8) 0.3478	(1) 0.0625	0.0915
	rs41511151	c.2078 G>A	(2) 0.0869	0	0.0119
FN1	rs115937626	c.40 G>C	(1) 0.0435	0	0.0000
	rs1250209	c.6415 G>A	(23) 1	(16) 1	1
	rs2577301	c.2449 A>C	(23) 1	(11) 0.6875	1
	rs1250259	c.44 A>T	(21) 0.9130	(14) 0.875	0.7674
ICAM1	rs5491	c.167 A>T	(1) 0.0435	0	0.0070
	rs5498	c.1405 A>G	(15) 0.6522	(13) 0.8125	0.4662
	rs1799969	c.721 G>A	(1) 0.0435	0	0.1213
	rs1801714	c.1055 C>T	(2) 0.0869	(3) 0.1875	0.0219
	rs376526495	c.1589 C>T	(1) 0.0435	0	0.0000
ITGB3	rs5918	c.176 T>C	(5) 0.2174	(6) 0.375	0.1322
	rs15908	c.1108 A>C	(18) 0.7826	(10) 0.625	0.3678
	rs121918449	c.1199 G>A	(2) 0.0869	0	0.000
	rs73322311	c.2173 C>T	(1) 0.0435	0	0.0000
	rs367659742	c.2351 C>T	(1) 0.0435	0	0.0010
LAMC1	rs20563	c.1372 A>G	(19) 0.8261	(2) 0.125	0.5636
	rs148690613	c.2053 A>G	(1) 0.0435	0	0.0010
	rs20558	c.2663 T>C	(19) 0.8261	(11) 0.6875	0.5636
	rs61749262	c.3614 A>G	(2) 0.0869	0	0.0159
	rs34995260	c.3796 G>A	(1) 0.0435	0	0.0060
LPAR4	rs148919808	c.133 G>A	(1) 0.0435	0	0.007
ROCK2	rs2230774	c.1292 C>A	(14) 0.6087	(10) 0.625	0.5268
SELE	rs5368	c.1402 C>T	(4) 0.1739	(1) 0.0625	0.1262
	rs5366	c.1261 G>C	(1) 0.0435	0	0.0000
	rs5361	c.445 A>C	(4) 0.1739	(3) 0.1875	0.0984
TNC	rs13321	c.6022 G>C	(22) 0.9565	(14) 0.875	0.7207
	rs2274750	c.5341 G>A	(1) 0.0435	0	0.0318
	rs61734387	c.5093 G>C	(2) 0.0869	(1) 0.0625	0.0129
	rs2104772	c.5029 A>T	(13) 0.5652	(11) 0.6875	0.4503
	rs113301777	c.3739 C>A	(4) 0.1739	(1) 0.0625	0.0179
	rs1138545	c.3197 G>A	(4) 0.1739	(1) 0.0625	0.1501
	rs1061494	c.2039 A>G	(17) 0.7391	(16) 1	0.4423
	rs1757095	c.1616 A>G	(23) 1	(16) 1	0.9404
VCAM1	rs3783613	c.1052 G>C	(1) 0.0435	0	0.0000
	rs3783615	c.1960 A>T	(1) 0.0435	0	0.0000

References

1. Bierzynska A, Soderquest K, Dean P, et al. MAGI2 mutations cause congenital nephrotic syndrome. *J Am Soc Nephrol*. **2017**;28(5):1614-1621. doi:10.1681/ASN.2016040387

2. Assad L, Schwartz MM, Virtanen I, Gould VE. Immunolocalization of tenascin and cellular fibronectins in diverse glomerulopathies. *Virchows Arch B Cell Pathol Incl Mol Pathol*. **1993**;63(1):307-316. doi:10.1007/BF02899277

3. Maxwell PH, Wang Y. Genetic studies of IgA nephropathy. *Nephron - Exp Nephrol*. **2006**;102(3-4):76-80. doi:10.1159/000089685

4. Adler S, Brady HR. Cell adhesion molecules and the glomerulopathies. *Am J Med*. **1999**;107(4):371-386. doi:10.1016/S0002-9343(99)00233-8
